# Occurrence and removal of phenolic endocrine disrupting chemicals in the water treatment processes

**DOI:** 10.1038/srep22860

**Published:** 2016-03-08

**Authors:** Xuemin Lv, Sanhua Xiao, Gang Zhang, Pu Jiang, Fei Tang

**Affiliations:** 1 Department of Environmental Microbiology, Institute of Environmental Medicine, MOE Key Lab of Environment and Health, School of Public Health, Tongji Medical College, Huazhong University of Science and Technology, Wuhan 430030, P.R. China

## Abstract

This paper evaluated the occurrence and removal efficiency of four selected phenolic endocrine disrupting chemicals (bisphenol A (BPA), octylphenol (OP), nonylphenol (NP) and diethylstilbestrol (DES)) in two drinking waterworks in Jiangsu province which take source water from Taihu Lake. The recombined yeast estrogen screen (YES) and liquid chromatography tandem mass spectrometry (LC-MS/MS) were applied to assess the estrogenicity and detect the estrogens in the samples. The estrogen equivalents (EEQs) ranged from nd (not detected) to 2.96 ng/L, and the estrogenic activities decreased along the processes. Among the 32 samples, DES prevailed in all samples, with concentrations ranging 1.46–12.0 ng/L, BPA, OP and NP were partially detected, with concentrations ranging from nd to 17.73 ng/L, nd to 0.49 ng/L and nd to 3.27 ng/L, respectively. DES was found to be the main contributor to the estrogenicity (99.06%), followed by NP (0.62%), OP (0.23%) and BPA (0.09%). From the observation of treatment efficiency, the advanced treatment processes presented much higher removal ratio in reducing DES, the biodegradation played an important role in removing BPA, ozonation and pre-oxidation showed an effective removal on all the four estrogens; while the conventional ones can also reduce all the four estrogens.

Endocrine disrupting compounds (EDCs) has been reported for over 80 years^1^. They are known as a class of chemicals which have xenobiotic and exogenous origins while mimicking or inhibiting the natural actions of the endocrine system in animals and human, such as synthesis, secretion, transport, and binding. They maintain the homeostasis, reproduction, metabolism, development, and/or behavior of living species and include diverse groups of heterogeneous contaminants (alkylphenols, polychlorinated biphenyls, selected pesticides, steroid sex hormones, phthalates etc.)^2^,^3^,^4^.

The presence of EDCs in the aquatic systems (wastewaters, drinking water and underground water) is considered as a major environmental issue^5^,^6^,^7^,^8^,^9^. Phenolic estrogens are commonly included in the reported EDCs in the wastewater and drinking water, such as bisphenol-A (BPA), diethylstilbestrol (DES), octylphenol (OP), nonylphenol (NP) and corresponding ethoxylates^10^,^11^,^12^. These compounds has become ubiquitous in the environment because of their presence in a multitude of products including food and beverage packaging, surfactants, flame retardants, adhesives, building materials, electronic components, paper coatings and pharmaceutical personal care products (PPCPs). They can cause adverse effects to human bodies^12^,^13^,^14^,^15^. Furthermore, the increasing incidences of cancer and decreasing reproductive fitness in humans are thought to be caused by exposure to estrogenic compounds, including phenolic compounds, especially via drinking water^16^,^17^. The occurrence and distribution of these phenolic EDCs have been widely reported in drinking water^18^,^19^,^20^, but limited studies focus on the removal of these chemicals during the drinking water treatment processes^21^,^22^, especially the situations in China.

As one of the most important drinking water sources, Taihu Lake supplies more than 50% of the population in Jiangsu province. Water quality in this area is attracting great environmental, health and safety concern. The occurrence of contaminants like EDCs has been reported as an increasing amount of pollutants that has been discharged into the lake directly and indirectly with the development of industry and agriculture^23^. However, few studies have comprehensively dealt with the occurrence and removal of the phenolic EDCs during the water treatment processes which take source water from Taihu Lake.

Conventional water treatment processes (coagulation, sedimentation, sand filtration, and chlorination) have been used for decades, and with the pollution becoming more serious and the purpose of improving drinking water quality, advanced water treatment processes (ozonation, activated carbon and ultra-membrane filtration) have been applied in a waterworks (Taihu Lake source) five years ago. Therefore, it is important to evaluate the concentrations and fate of phenolic EDCs in the waterworks since the related studies on these compounds in drinking water systems are limited, and the efficiencies of advanced processes need to be evaluated.

The instrumental monitoring like liquid chromatography tandem mass spectrometry (LC-MS/MS) can provide qualitative and quantitative information about the environmental contaminants in the water samples, while bioassays can provide measures of the cumulative effects of chemicals that exhibit the same mode of toxic action and thus concentration additive effects. So bioassays are usually used as a complement to instrumental analyses in toxicity identification evaluation schemes to address complex environmental issues^24^. Therefore, in this paper, LC-MS/MS was applied to detect the four selected EDCs (nonylphenol, octylphenol, bisphenol A and diethylstilbestrol) and the *in vitro* bioassay (recombined yeast estrogen screen, YES) was employed to assess the estrogenic activities at the same time.

Thus the objectives of this paper were to (a) determine the occurrence of the phenolic EDCs in two waterworks that both take source water from Taihu Lake, (b) to discuss the removal efficiencies of the target phenols in the conventional water treatment processes and the advanced treatment processes, (c) to find out the main contributor to the estrogenicity of the four target phenols.

## Methods

### Reagents and chemicals

Chemicals used in the YES and standard substances in the chemical analysis were purchased from Sigma-Aldrich Chemical Co. (St. Louis, MO USA): Chlorophenol Red-β-D galactopyranoside (CPRG), 17β-estradiol (E2), nonylphenol (NP), octylphenol (OP), bisphenol A (BPA), bisphenol A-d_16_ and diethylstilbestrol (DES). All reagents of HPLC grade used for sample processing and analysis (methanol, dichloromethane, n-hexane and acetone) were obtained from J.T.Baker Avantor Co. (Center Valley, PA, USA). Glass fiber filters (GF/F, pore size 0.7 μm) were obtained from Whatman (Maidstone, UK) and prebaked at 450 °C for 4 h prior to use. Deionized organic-free water was obtained from a Milli-Q water purification system from Millipore (Bedford, USA). The solid-phase extraction sorbent Sep-Pak C18 vac cartridge (1000 mg, 6 cc) was supplied by Waters Co. (Milford, MA, USA). Stock solutions of the four phenols were prepared in methanol at 1 μg/μL and stored at −20 °C for later use; the appropriate working standard solutions were prepared in methanol and stored in amber glass bottles at 4 °C.

### Sampling and preparation of solid-phase extraction

**Figure 1:**
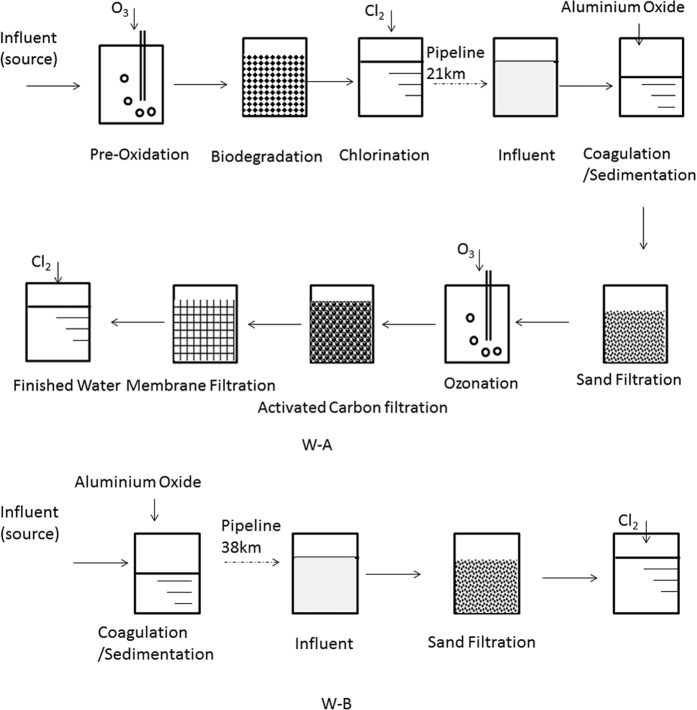
The water treatment processes of waterworks A (W-A) and waterworks B (W-B).

Influent (raw water) and effluent (of each treatment process) samples were collected from two waterworks (W-A applied advanced water treatment processes and W-B applied the conventional ones). Sampling campaigns were performed in January and March 2014 that were both during the same hydrographic period (dry period). Scholars reported that in the dry period (December to March) the water level was relatively low and the pollution level is relatively high in Taihu lake, and the estrogencity was more intense compared with other periods^25^, so the results can be more representative in reflecting the occurrence and removal of the of the phenols during the different water treatment processes. Additional details regarding these waterworks are given in Table 1 and Fig. 1.

The samples (4 L each) were collected in amber glass bottles, and filtered through prebaked glass fiber filters to avoid extraction cartridges clogging^11^. The solid phase extractions were performed on the Visiprep™ SPE Vacuum Manifold DL, for 24 samples from Supelco (Bellefonte, PA, USA). The SPE C18 cartridges, prior to extraction were conditioned with 5 mL methanol and 5 mL deionized water. Water samples were passed through the cartridges at a flow rate of 5 mL/min under vacuum. After dried under vacuum for 1 h, the target compounds were eluted with 2 mL methanol, followed by 2 mL dichloromethane and 2 mL n-hexane. Then the extracts were dried under a gentle nitrogen stream at 45 °C. For the bioassay (YES), these extracts were dissolved in the anhydrous ethanol to make 0.1 mL solutions presented for 250 mL water sample extracts,viz.,the concentration factor is 2500; for chemical analysis (LC-MS/MS) the extracts were dissolved in methanol to make 2000 fold concentration. Each final extract was then filtered through a 0.22 μm membrane filter into a 2 mL sealed amber glass vial. The vials were kept at −20 °C for later analysis. Blank columns were extracted and treated in the same way as described above.

### Yeast estrogen screen (YES) bioassay

The recombinant yeast estrogen screen was carried out according to the protocols detailed by Routledag and Sumpter^26^ using a slightly modified version of procedure described by Rastal *et al.*^27^. Briefly, the recombinant yeast cells were stably transfected with the gene of the human estrogen receptor (hER) and containing expression plasmids carrying strong promoter sequences and the lac-Z (β-galactosidase) reporter gene. Using appropriate growth media, the recombinant yeast cells express hER in a form capable of binding to estrogen response elements (ERE) situated within a promoter sequence on the plasmid. Following the binding of a suitable agonist, the agonist-hER complex interacts with various transcription factors, binds to the ERE and initiates a cascade of events which results in the expression of the lac-Z reporter gene and the secretion of β-galactosidase into the assay medium.

The briefly assay procedure: three 100 μL extracts representing 250 mL water sample were serially diluted along alternate rows of a 96 well microtitre plate. A 17β-estradiol (E2) positive control was added to a separate row and serially diluted to give a final concentration range of 1.00 × 10^–8^ to 4.80 × 10^–12 ^mol/L. 50 mL of assay medium containing 500 μL of a 1.65 × 10^–2 ^mol/L aqueous solution of the chromogenic substrate Chlorophenol-Red-β-D-galactopyranoside (CPRG) and 4.0 × 10^7^^ ^/L recombinant yeast cells were then prepared and 200 μL transferred to each well. The plates were sealed and incubated at 32 °C for 72 h. Estrogenic potential was subsequently determined photometrically at 540 nm following the conversion of the CPRG from yellow to red by β-galactosidase secreted into the growth medium in response to the presence of hER agonists in the sample^28^.

### Instrumental analysis

The target compounds were analyzed by reversed phase liquid chromatography (Agilent 1260 Infinity, Agilent Technologies, Santa Clara, USA) coupled to a triple quadrupole (QqQ) mass spectrometer (LC-MS/MS 6460, Agilent Technologies, Santa Clara, USA) equipped with an electrospray ionization interface (ESI). The apparatus was composed of a binary pump, autosampler, a degasser and temperature controlled column compartment. The chromatograms were recorded and evaluated by means of the Mass Hunter Workstation Acqusition (Agilent Technologies, Santa Clara, USA). Chromatographic separation was performed on a Zorbax Eclipse Plus C18 column (100 mm × 2.1 mm i.d., particle size 3.5 μm, Agilent Technologies, Santa Clara, USA). Nitrogen was used as the collision and nebulizing gas.

To improve the separation and reduce the analysis time, different experiments were carried out in the gradient mode, the optimal gradient conditions were as follows: Gradient elution consisted of a solution of 30: 70 (v/v) water : methanol started from 0 minute, followed by an increase in methanol to 80% in 1 minute; held for half a minute; from 1.5 to 2 min methanol increased to 100%; held for two and a half minutes, finally, from 4.5 to 5 min methanol decreased back to 70%. The injection volume was 5 μL and the flow rate was 0.25 mL/min. The temperature in the column compartment was set at 40 °C. The analysis was performed using ESI negative, and target analytes were identified and quantified using the multiple-reaction monitoring (MRM) mode. The retention time and MRM parameters are summarized in Table 2.

The estrogen equivalent concentrations measured by the YES assay (EEQs_(bio)_, referring estrogenic activity to the natural estrogen 17β-estradiol^29^,^30^) were determined as follow: Estrogenic activities of water extracts are expressed as a percentage of the maximum response of the positive control E2 from the same microtiter plate and are plotted to logarithmically transformed equivalents of water sample absolute per well^31^. Also the EEQs were calculated from the chemical analysis of the target compounds based on the theory that all the investigated chemicals target the same receptor^32^,^33^. The calculated EEQs (EEQs_(chem)_) were expressed as the sum of all estrogenic contributions of four compounds by multiplying their estradiol equivalent factors (EEFs) (Table 2) and chemical concentration.

### Quality control and quality assurance

All data were subject to strict quality control procedures. The linearities of the method were tested with calibration standards at five concentration levels ranging from 0.01 to 100 μg/L. Good linearities were observed for the four target phenolic EDCs, with all the correlation coefficients (*r*^*2*^) above 0.99. Limits of quantification (LOQs) (signal-to-noise ratio = 10) and limits of detection (LODs) (signal-to-noise ratio = 3) of the four target compounds are shown in Table 2. The recovery studies were performed on 4 L of deionized organic-free water and 4 L of raw water collected from the Taihu Lake, spiked with known levels (final concentration 100 ng/L) of the four compounds respectively (three replicates). Mean recoveries ranged from 87.4% to 116.9% for all the targets in the two matrices. The 100 ng /L mixture of standards was analyzed for six times within a day. The relative standard deviations (RSD) of concentrations for all the targets ranged from 3.19% to 7.15% (Table 3), demonstrating the high precision of the analysis methods. In order to ensure the accuracy of the analysis, all samples were replicated three times, and the final concentration averages used.

For the ubiquity of the phenols and to avoid contamination during the sampling and experiment processes, sampling bottles and all glassware used in the experiment were cleaned by washing with detergent, rinsed with deionized organic-free water, and baked at 450 °C for at least 4 h. All laboratory materials were either made of glass or Teflon to avoid sample contamination.

## Results and Discussion

### Estrogenic activities of the samples in YES assay

All extracts of the water samples were found to induce estrogenic activities at different levels (Table 4). The EEQs_(bio)_ values ranged from below LOD to 2.96 ng/L. The estrogenic activities of March samples are a little higher than the samples collected in January, maybe the temperature gets warmer and the activities of the residents around the lake become more frequency lead to these changes. Both the two waterworks showed a good efficiency in reducing the estrogenic activities, for the estrogenic activities decreased along the processes in both waterworks, and the estrogenicites of the finished water are much lower compared to the relative raw water. According to NOEC (no observed effect concentration) of estrogenicity calculated by the researches in the *in vitro* test^34^,^35^, EEQs_(bio)_ <0.4 ng/L was considered as the toxic threshold to human being. Thus in our study, except for the raw water of waterworks B, the estrogenic activities of rest water samples were lower than the threshold. We could also find that the estrogencities of the samples were lower in waterworks A, considering the different water intake points of the two waterworks, water works B is much nearer to the tributary that run through the residential districts which may bring in more contaminants, that may be the cause to the different estrogenicities.

The *in vitro* estrogenic activities of the raw waters were almost at the same level compared to home and abroad, where the estrogenic activities of the Taihu Lake ranged from 2.2–8.3 ng/L in China, 0.30–4.50 ng/L of Seine River in France, 0.3–7.0 ng/L in rivers of Switzerland and 0.7–4.01 ng/L in Tokyo Bay of Japan^36^,^37^,^38^,^39^.

### Occurrence of target phenolic compounds in influent and effluent samples

**Figure 2:**
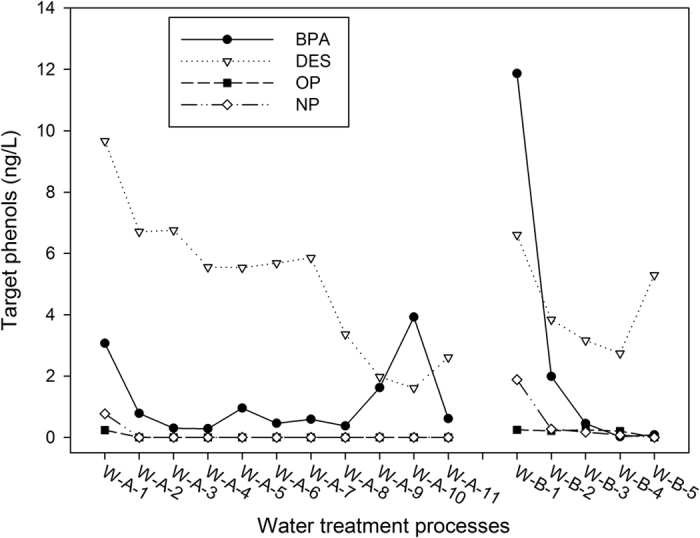
The occurrences of the selected compounds in the water treatment processes. W-A-1: Influent (source); W-A-2: Pre-Oxidation; W-A-3: Biodegradation; W-A-4: Chlorination; W-A-5: Influent; W-A-6: Coagulation and Sedimentation; W-A-7: Sand Filtration; W-A-8: Ozonation; W-A-9: Activated Carbon filtration; W-A-10: Membrane Filtration; W-A-11: Finished Water. W-B-1: Influent(source); W-B-2: Coagulation and Sedimentation; W-B-3: Influent; W-B-4: Sand Filtration; W-B-5: Finished Water.

The occurrences of the selected compounds in influent and effluent samples are shown in Table 4 and Fig. 2. Among the 32 samples, DES prevailed in all samples, with concentrations ranging 1.46–12.0 ng/L. BPA, OP and NP were partially detected with concentrations ranging from nd (<LOD, cannot be detected) to 17.73 ng/L, nd to 0.49 ng/L and nd to 3.27 ng/L, respectively. In general, concentrations of BPA and DES were much higher than the other two compounds; OP was detected in five samples. Regarding the relative regulations and acts, China government sets limited of BPA in drinking water as below 0.01 mg/L^40^ and European Commission identified NP as priority hazardous substance^41^; Other EDCs are rarely seen the threshold or limit. These emerging compounds appeared a few decades ago, and the toxicity need to be studied and understand further, the relative regulations also need to be revised as the research being further, there were studies pointed out that BPA could cause adverse effects at very low dose that far below the regulations^42^. Therefore, more attention should be paid to the occurrence of these chemicals.

The variation trend of the four target phenols was shown in Fig. 2. In general, the four chemicals are decreased along the treatment processes, and NP and OP are rarely detected after the raw water being treated. In waterworks A, the concentration of BPA goes up a little after the membrane filtration step; and DES was raised a bit in both the two waterworks in the final treatment processes. The phenomenon was discussed in the later parts.

In the work of Jiang *et al.*, concentrations of OP, NP and BPA detected in Taihu Lake were 3.08–89.52 ng/L; 30.09–280.19 ng/L and 7.61–710.65 ng/L, respectively^20^. These results are similar to those determined in our study. The concentrations of BPA, DES, OP and NP in the lake water of Wuhan were 20–534 ng/L, nd–9 ng/L, 10–133 ng/L and 260–4042 ng/L, respectively^12^, the observed concentrations of OP, NP and BPA in Wuhan were remarkably higher than our study except for the DES. The different turn out may be due to the different water body and in Wuhan the runoff nearby the lake rarely cover industrial and agricultural activities, the runoff are mostly from the pollutants of human daily activities. All these researches manifested that the drinking water source had been polluted by the phenols at different levels.

### Estrogenic activities calculated from the chemical analysis

In order to compare the results of the biological and chemical analysis and to estimate the contribution of target analytes to the overall estrogenic activities of the real samples, chemical estradiol equivalents (EEQs_(chem)_) were determined. Therefore, concentration data obtained by LC–MS/MS analysis were multiplied with the determined relative potencies expressed as EEFs of the compounds in the YES. The EEFs were determined following the principle introduced by Beck^31^, and the EEFs of the four selected chemicals are shown in Table 2. The EEQs_(chem)_ are listed in Table 3. From the table, we could find the EEQs vary in the same trend. But there exist some variations, such as the EEQs_(bio)_ of source water are much higher than the relative EEQs_(chem)_ especially the samples of W-B; and for some samples, the EEQs_(bio)_ cannot be calculated for the negative reactions in the bioassay while some of the selected compounds can stilled be detected by LC-MS/MS.

The differences between EEQs_(bio)_ and EEQs_(chem)_ could have diverse reasons. In general, both the variability of the biological test system and the uncertainty of trace analysis in the ng/L range on the equipment could contribute to the deviations. Significantly higher values obtained in the YES indicated that there were estrogenic compounds in the samples, which were not detected by the chemical target analysis. Reports on the estrogenic activities of surface water pointed out that the steroid estrogens were another group of chemicals that contribute to the estrogenicities^43^,^44^,^45^,^46^,^47^ and Korner, W. *et al.*^48^ suggested that E2 and EE2 were the major contributing substances to estrogenic activity in water samples. Anti-estrogenic activities could be the reason for the lower EEQs measured with the YES. This comparison underlines the difficulties in achieving consistent results from biological and chemical analysis, especially in the case of complex environmental samples.

The contributions of the four phenols to the whole sample estrogenic activities were determined as follow: the EEQs_(chem)_ of each phenol divided the sum of EEQs_(chem)_ of the four. The contribution ratio to the estrogenic activities induced by the four target phenolic compounds were: DES (99.06%), followed by NP (0.62%), OP (0.23%) and BPA (0.09%); therefore, DES was the mainly contributor to the estrogenicity induced by the four phenols.

### Removal efficiencies of phenolic EDCs in conventional and advanced water treatment processes

**Figure 3:**
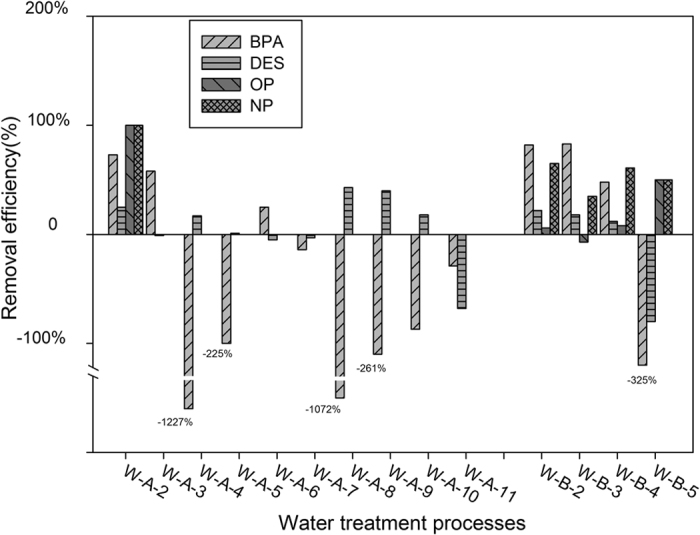
The occurrences of the selected compounds in the water treatment processes. W-A-2: Pre-Oxidation; W-A-3: Biodegradation; W-A-4: Chlorination; W-A-5: Influent; W-A-6: Coagulation and Sedimentation; W-A-7: Sand Filtration; W-A-8: Ozonation; W-A-9: Activated Carbon filtration; W-A-10: Membrane Filtration; W-A-11: Finished Water. W-B-2: Coagulation and Sedimentation; W-B-3: Influent; W-B-4: Sand Filtration; W-B-5: Finished Water. Use 0 to replace nd when calculating. The removal efficiency of each process is calculated as follows: (C_0_−C_1_)/C_0_ × 100% C_0_: the concentration of the target compounds of process_0_ C_1_: the concentration of the target compounds of process_1_ (the process following process_0_).

The main goal of this work was to provide important insight into the technology that can most effectively remove phenolic EDCs in the water treatment. The removal efficiencies in each process of the two waterworks were individually analyzed in this study (Fig. 3), as described below.

Total removal efficiencies of each waterworks were also calculated as the concentration of finished water compared to the raw water; the results were: Waterworks A: BPA: 83.34%, DES 64.36%, NP100%, OP 100%. Waterworks B: BPA: 99.52%, DES: 13.45%, NP100%, OP 100%.

### Removal of BPA

Though BPA is the weakest estrogen among the four selected phenolic compounds according to the EEF values (Table 2) and BPA contributed least according to the contribution ratio, both the two waterworks showed good efficiencies (W-A 83% and W-B 99%) in removing BPA from the total treatment processes. The conventional treatment processes (coagulation, sedimentation and sand filtration) present high removal efficiencies, after these treatments, BPA was reduced to the detection limit in some processes, which is in accordance with the researches^49^,^50^. Also the pre-oxidation and biodegradation in advanced processes were remarkable in removing BPA with the efficiency 73% and 58%, just like the study pointed out^51^. However, after the membrane filtration step in waterworks A, the concentration of BPA increased (Table 3), considering the application of BPA as a plasticizer in the plastic manufacturing, the leaching of BPA from the ultrafiltration membrane may be the cause^52^. From our study, both conventional and advanced water treatment processes can effectively reduce the concentration of BPA despite the concentration may be increased in some intermediate processes. The removal efficiencies in Fig. 3 like −1227%, −225% and −1072% etc., these values indicating the increasing of BPA and because of the relative low concentration in the former process, the values were much higher when the concentration increased a little.

### Removal of DES

Although limited studies focus on the DES in water, DES presents relative high estrogenic activity as the EEF is the highest among the four phenolic compounds, and Jin *et al.* pointed that DES presented a high risk in source water^12^. In this study, DES presented the highest concentration and the highest contribution ratio, so the people whose drinking water source from the lake are at high risk. The high dose of DES presented in the drinking water may be caused by the drug abuse, for DES was synthetic and used in estrogen replacement therapy. W-A applying the advanced water treatment shows a good removal with the total efficiency 64%, while W-B, the conventional treatment processes is not so efficient as the efficiency 13%. Through all the conventional treatment technologies, the removal efficiencies are all around 20%, while the advanced ones shows a much higher ratio as the pre-oxidation, ozonation, activated carbon filtration and membrane filtration presented the efficiencies over 20% even above 40%. The advanced oxidation processes like pre-oxidation and ozonation that can effectively reduce the concentration of DES may due to the chemical characteristic that DES is easily to be oxidized. As presented in our study, advanced treatment processes show a more remarkable removal than the conventional ones.

### Removal of OP and NP

Chen *et al.* reported that oxidation plays an important role in the degradation of NP in water treatment processes^49^. As presented in our study, the pre-oxidation can remarkably reduce the concentration of OP and NP with both the efficiencies are 100%, indicating that oxidation may be a good way to control EDCs in the water^21^. However, both the two waterworks can effectively reduce the concentration of OP and NP to below detection, meaning conventional treatment processes were capable in controlling OP and NP levels in finished water even without oxidation.

All these target four phenolic compounds (BPA, DES, NP and OP) tend to bond with suspended particles due to their high K_ow_ values and can thus be partially removed by coagulation, sedimentation and sand filtration, just in accordance to many researches^21^,^49^,^53^ and as shown in this study, these processes can also remove the estrogens to different extend. It is known that phenolic groups can be oxidized by strong oxidizers, so pollutants containing them, as it is the case of phenolic estrogens, can undergo transformation during chlorination and ozonation^54^,^55^, and in our study the oxidation processes showed satisfied removal efficiencies. The biodegradation process is another important part of the advanced water treatment processes as it is the process by which microbial organisms transform or alter (through metabolic or enzymatic action) the structure of chemicals introduced into the environment^56^, just as presented in this study, the biodegradation process can effectively reduce BPA.

### Overall removal capacity of the conventional and advanced treatment processes

As mentioned above, the total treatment removal efficiency manifested that the conventional and advanced treatment processes can effectively reduce the concentration of BPA, NP and OP with the removal efficiencies were above 80% even to 100%. However, regarding the case of DES, advanced treatment processes showed much higher reducing ratio to the conventional processes (64.36% to 13.45%); generally speaking, the advanced oxidation processes like pre-oxidation and ozonation can remarkably reduce the contaminants. Thus considering the pollution situations of the drinking water source, the advanced treatment processes is recommended.

## Conclusion

In this study, YES assay showed that the estrogenic activities decreased along the processes in both conventional and advanced treatment processes, and the LC-MS/MS analysis found out that BPA, DES and NP were identified in most of the samples, and DES was identified to be the main contributor to the estrogenicity among the four target compounds. From the observation of treatment efficiencies, both conventional and advanced treatment processes can effectively reduce the concentration of BPA, OP and NP, while the advanced ones presented much higher removal ratio in reducing DES. The biodegradation plays an important role in removing BPA and ozonation and pre-oxidation show an effective removal on BPA, DES, OP and NP. Thus a conclusion can be drawn that the advanced water treatment processes were more effective in removing the four phenolic compounds. However, it should be noted that treatment efficiency with low level of chemicals cannot be justified properly, because its concentrations were close to the detection limit. Finally, more specific study seems to be necessary to further understand the mechanisms of the fate and transport of EDCs (phenols, steroids and phthalates) during both conventional and advanced treatment processes.

## Additional Information

**How to cite this article**: Lv, X. *et al.* Occurrence and removal of phenolic endocrine disrupting chemicals in the water treatment processes. *Sci. Rep.* **6**, 22860; doi: 10.1038/srep22860 (2016).

## Figures and Tables

**Table 1:** Details of the waterworks studied.

Waterworks	Capacity^a^	Treatment Process	Additional Information^a^
W-A	1.0 Million ton	Influent (source)	Raw Water From Taihu Lake
		Pre-Oxidation	Ozonation: 0.6 mg/L
			Gas Water Ratio: 0.6–0.8: 1
			Contact Time: 10 minutes
		Biodegradation	Biological Aerated Filter
			Working Depth = 2.0 m
			Gas Water Ratio: 0.6–0.8: 1
		Chlorination	Residual Chlorine 0.05 mg/L
		Influent	Pipeline length: 21 km
			Pipeline Retention Time: 6–7 h
		Coagulation and	Aluminium Oxide: 30 mg/L
		Sedimentation	Contact Time: 3–4 h
		Sand Filtration	Quartz Sand: Pore Size 0.75 mm H = 70 cm
			Flow Rate: 5 ~ 6 m/h
		Ozonation	Ozonation: 0.8 mg/L
			Contact Time: 10–15 min
		Activated Carbon filtration	Granular Activated Carbon: *d *= 1.5 mm; *H *= 2.1 m, *S *= 0.5 × 0.5 m
		Membrane Filtration	Polyvinylidene Fluoride(PVDF) Member: 0.45 μm
		Finished Water	Residual Chlorine 0.8 mg/L
			Retention Time 3 h
W-B	0.5 Million ton	Influent(source)	Raw Water From Taihu Lake
		Coagulation and	Aluminium Oxide: 50 mg/L
		Sedimentation	Contact Time: 2 h
		Influent	Pipeline length: 38 km
			Pipeline Retention Time: 12–15 h
		Sand Filtration	Quartz Sand: Pore Size 0.75 mm H = 70 cm
			Flow Rate: 5 ~ 6 m/h
		Finished Water	Residual Chlorine 0.4–0.6 mg/L
			Retention Time 2–3 h

^a^design values.

**Table 2:** Retention time, MRM parameters, LOQs and LODs of target compounds.

Compounds	RT(min)	Precursor ion(m/z)	Compouns parameter		Product ion	LOQ(ng/L)	LOD(ng/L)	EEF
			Fragmentor (V)	Collision energy (V)				
Bisphenol A (BPA)	1.92	227	60	20/15	212*/133	11.3	3.4	3.0 × 10^−5^
Diethylstilbestrol (DES)	2.40	262	60	30/30	237*/222	7.7	2.3	2.1 × 10^−2^
Octylphenol (OP)	4.39	205	60	15	106	28.0	8.4	1.2 × 10^−3^
Nonylphenol (NP)	4.60	219	60	15	106	30.7	9.2	7.0 × 10^−4^

RT: retention time *quantification ion Source parameter: gas temperature: 300 °C, gas flow: 5 L/min, Nebulizer: 45 psi, sheath gas temperature: 250 °C, sheath gas flow: 11 L/min, capillary (negative) 3500 V, nozzle voltage 500 V.

**Table 3:** Mean percentage recovery and relative standard deviations (RSD) of target substances.

Compounds	Recovery (%)^a^	Recovery (%)^b^	RSD (%)
Bisphenol A (BPA)	116.9	98.5	7.15
Diethylstilbestrol (DES)	87.4	102.1	5.36
Octylphenol (OP)	91.4	87.6	3.42
Nonylphenol (NP)	92.3	95.3	3.19

^a^recovery in organic-free water.

^b^recovery in raw water.

**Table 4:** The estrogenicity (EEQs, mean ± deviation) and the concentration of the four selected phenolic estrogens (ng/L).

Waterworks	Treatment Process	January 2014						March 2014					
		BPA	DES	OP	NP	EEQs(bio)	EEQs(chem)	BPA	DES	OP	NP	EEQs(bio)	EEQs(chem)
W-A	Influent (source)	3.66	7.33	0.48	0.29	0.20±0.05	0.15	2.47	12.0	nd	1.25	0.21±0.06	0.25
	Pre-Oxidation	0.72	6.74	nd	nd	nd	0.14	0.85	6.68	nd	nd	0.29±0.02	0.14
	Biodegration	0.6	7.21	nd	nd	0.08±0.02	0.15	nd	6.31	nd	nd	0.23±0.02	0.13
	Chlorination	0.32	5.41	nd	nd	nd	0.11	0.25	5.69	nd	nd	0.28±0.05	0.12
	Influent	1.30	4.64	nd	nd	nd	0.10	0.61	6.42	nd	nd	0.33±0.05	0.13
	Coagulation and Sedimentation	0.01	5.37	nd	nd	0.03±0.05	0.11	0.91	5.99	nd	nd	0.22±0.01	0.13
	Sand Filtration	0.01	5.75	nd	nd	nd	0.12	1.17	5.96	nd	nd	0.22±0.01	0.13
	Ozonation	0.23	2.90	nd	nd	0.05±0.04	0.06	0.52	3.82	nd	nd	0.15±0.01	0.08
	Activated Carbon filtration	0.41	1.91	nd	nd	0.08±0.02	0.04	2.83	2.04	nd	nd	nd	0.04
	Membrane Filtration	0.47	1.76	nd	nd	0.07±0.01	0.04	7.37	1.46	nd	nd	nd	0.03
	Finished Water	1.22	1.92	nd	nd	0.07±0.01	0.04	nd	3.30	nd	nd	0.09±0.09	0.07
W-B	Influent(source)	17.73	3.21	0.49	0.50	1.47±0.06	0.07	6.00	10.0	nd	3.27	2.96±0.13	0.21
	Coagulation and Sedimentation	2.76	3.76	0.43	0.31	0.06±0.06	0.08	1.21	3.92	nd	0.23	1.00±0.02	0.08
	Influent	0.88	3.31	0.49	0.19	0.02±0.04	0.07	0.02	3.01	nd	0.16	0.22±0.04	0.06
	Sand Filtration	0.02	2.11	0.41	0.15	0.05±0.04	0.04	0.02	3.37	nd	nd	0.26±0.02	0.07
	Finished Water	0.17	2.59	nd	nd	0.02±0.03	0.05	nd	8.00	nd	nd	0.20±0.01	0.17

nd: cannot be detected, below LOD.
